# Impact of Dose Tapering of Tumor Necrosis Factor Inhibitor on Radiographic Progression in Ankylosing Spondylitis

**DOI:** 10.1371/journal.pone.0168958

**Published:** 2016-12-29

**Authors:** Jun Won Park, Hyun Mi Kwon, Jin Kyun Park, Ja-Young Choi, Eun Bong Lee, Yeong Wook Song, Eun Young Lee

**Affiliations:** 1 Division of Rheumatology, Department of Internal Medicine, Seoul National University College of Medicine, Seoul, Republic of Korea; 2 Department of Radiology, Seoul National University Hospital, Seoul, Republic of Korea; Oregon Health and Science University, UNITED STATES

## Abstract

**Objective:**

To investigate the impact of dose reduction of tumor necrosis factor inhibitor (TNFi) on radiographic progression in ankylosing spondylitis (AS).

**Methods:**

One hundred and sixty-five patients treated with etanercept or adalimumab were selected from a consecutive single-center observational cohort based on the availability of radiographs at baseline and after two- and/or four-years of follow up. Radiographs were assessed by two blinded readers using the modified Stokes AS Spinal Score (mSASSS). Radiographic progression in patients treated with standard-dose TNFi (standard-dose group, n = 49) was compared with patients whose dosage was tapered during the treatment (tapering group, n = 116) using linear mixed models.

**Results:**

Baseline characteristics between two groups were comparable except for higher BASDAI (7.1 vs. 6.3, p = 0.003) in the standard-dose group. At two years after the treatment, mean dose quotient (S.D.) of the tapering group was 0.59 (0.17). During follow up, rate of radiographic progression in overall patients was 0.90 mSASSS units/year. Radiographic progression over time between the two groups was similar at the entire group level. However, in the subgroup of patients with baseline syndesmophytes, progression occurred significantly faster in the tapering group after the adjustment for baseline status (1.23 vs. 1.72 mSASSS units/year, p = 0.023). Results were consistent when radiographic progression was assessed by the number of newly developed syndesmophytes (0.52 vs. 0.73/year, p = 0.047). Sensitivity analysis after multiple imputation of missing radiographs also showed similar results.

**Conclusion:**

A dose tapering strategy of TNFi is associated with more rapid radiographic progression in AS patients who have syndesmophytes at baseline.

## Introduction

Ankylosing spondylitis (AS) is a chronic inflammatory rheumatic disease that mainly affects the axial skeleton such as the sacroiliac joints and spine. Its pathognomonic structural damage is the development of syndesmophytes; it progresses slowly and is closely associated with subjective symptoms, impairment in mobility and deterioration in functional status [1–3]. The current treatment guideline recommends the assessment of structural damage using conventional radiographs, which has been included in the Assessment of Spondyloarthritis international society (ASAS) core set [4].

The impact of tumor necrosis factor inhibitor (TNFi) on radiographic progression in AS is still under debate. Spinal inflammatory lesions on MRI were rapidly improved by TNFi but continuous treatment for two years failed to inhibit the new bone formation [5–8]. Conversely, some cohort studies suggested that early and/or long-term continuous use of TNFi showed a diminished radiographic progression [9, 10]. However, despite such controversy, TNFi has been the only option for AS patients who remain active after the first-line non-steroidal anti-inflammatory drug (NSAID) treatment until the introduction of an interleukin-17A blocking agent. Since discontinuing TNFi usually leads to clinical relapse within a short time, patients who started this agent are recommended to continue it, which can cause various adverse events and create a substantial economic burden [11–13]. Previous studies have reported that low-dose TNFi treatment effectively maintained low disease activity in patients with AS [14–16]. However, the impact of dose tapering on radiographic progression has not been investigated because most studies regarding this issue have relatively short timeframes insufficient to detect a structural change.

In our clinical setting, a tapering dose of self-injectable TNFi has been utilized for a long time, along with the standard-dose TNFi treatment. So it is suitable to directly compare the radiographic progression over time between the two treatment strategies. In the present study, we investigated the radiographic progression of AS patients using TNFi and analyzed its difference over time between the standard-dose and the tapering regimen in a single-center observational cohort during four years of follow up.

## Methods

### Study patients and clinical assessment

Data on AS patients extracted from a consecutive single-center observational cohort (SNUH-biologics cohort). This cohort included 361 AS patients who started etanercept or adalimumab between January 2004 and December 2014 in a tertiary referral center in South Korea. Among them, we recruited patients based on the availability of cervical and lumbar radiographs at baseline and after two and/or four years of the treatment.

All patients fulfilled the modified New York criteria for AS at diagnosis and started TNFi if they showed high disease activity (Bath Ankylosing Spondylitis Disease Activity Index [BASDAI] ≥ 4) despite using NSAIDs for more than three months [17]. Clinical monitoring was performed at baseline (time-point at starting TNFi), three months after the baseline visit, and each subsequent six months. Disease activity was assessed using BASDAI and serum C-reactive protein (CRP). All patients were monitored at each visit to continue the treatment based on fulfillment of BASDAI 50 response criteria [18]. Low disease activity was defined as BASDAI < 4 and CRP < 0.5mg/dL, based on previous reports [15, 19]. If a patient discontinued the TNFi or switched to other agents, observation was terminated.

Demographic and clinical features at baseline visit were obtained from patients’ medical record. Data on concomitant NSAID intake during the TNFi treatment was measured by the NSAID index and high NSAID intake was defined as NSAID index ≥ 50 as in a previous study [20, 21]. Time-averaged values of the BASDAI and CRP over 4 years (between the baseline and 45-month follow up) was also calculated. This study was carried out in accordance with the Helsinki Declaration and was approved by the institutional review board of Seoul National University Hospital (H1310-085-528). Patients consent was waived by the review board since the research presents no more than minimal risk of harm to the subjects and it would not adversely affect the rights and welfare of them. All data were anonymized prior to analysis.

### Strategy for dose adjustment of TNFi

Because this study was observational, there was no fixed protocol for dose adjustment of TNFi and it was mainly proposed according to the treating physician’s decision. However, in this study, patients who reduced the dosage (tapering group) started TNFi at a standard dosage and tapering was tried shortly after achieving low disease activity with standard-dose treatment. In fact, all patients in the tapering group first reduced the dosage within the 2 years of TNFi treatment. They could escalate the dosage temporarily if a disease flare was suspected.

For quantification of dose reduction of TNFi, we used the term ‘TNFi index’, which is calculated as (actual prescribed dose / standard dose) x (standard dosing interval / actual dosing interval). For example, a patient administered subcutaneous adalimumab 40mg every 2 weeks for 3 months and tapered to 40mg every 3 weeks for the next 3 months, TNFi index during this period was 0.83 [{(40mg/40mg) x (2 weeks/2 weeks) x (3 months/6 months)} + {(40mg/40mg) x (2 weeks/3 weeks) x (3 months/6 months)}]. Data on TNFi use was collected every six months during the follow up.

### Radiographic assessment

Radiographs were scored using the modified Stoke Ankylosing Spondylitis Spine Score (mSASSS) [22]. Two trained readers (PJW and KHM) independently scored radiographs, blinded to demographic and clinical factors except for the chronologic information because this method has a higher sensitivity to change [23]. If a radiograph had ≤ 3 missing vertebral corners (VCs), missing scores were substituted by the mean score of a corresponding segment. In cases with > 3 missing VCs, this set of radiographs was excluded.

For analysis of radiographic progression, the mean mSASSS of both readers were used. If a score differed between the two readers by more than five units (defined as a major disagreement), the radiographs were re-scored by the same two readers. In cases of persistent major disagreement after the reassessment, a senior reader (CJY) evaluated the image and gave the final score. In addition, the number of syndesmophytes per patient was used for the estimation of radiographic progression. Only VCs where both readers agreed to the presence of syndesmophytes were used.

### Statistical analysis

The Student’s t-test or Mann-Whitney U test was used for comparison of continuous data between the two groups. Inter-observer reliability was assessed using the intra-class correlation coefficient (ICC) for entire radiographs and smallest detectable change (SDC) for 2-year mSASSS progression. SDC is the smallest amount of progression that is reliably detectable above the measurement error and is calculated by ‘1.96*SD_Δ (progression score) / (√2*√k)_’ where k represents the number of readers [16]. Radiographic progression over time was evaluated using mixed model approaches. This statistical technique can provide a more flexible approach in handling unbalanced longitudinal data and allows other possible risk factors for radiographic progression to be included in the models [24]. In the modeling process, a ‘compound symmetry’ correlation structure was used and time was modeled in linear and non-linear (quadratic and cubic) modes. Fitness of models was assessed by Bayesian information criterion (BIC). Interaction between demographic and clinical factors and time were investigated and the analysis was repeated after stratification when significant interactions were found. To compare radiographic progression over time between the two groups, potentially related factors were identified first. These included age, gender, disease duration, BMI, smoking, baseline and time-averaged disease activities, HLA-B27, hip involvement, baseline mSASSS and NSAID index. Impact of each variable on radiographic progression over time was estimated (clinical factor x time interaction). The main multivariable model included only variables which showed potentially significant interaction (p <0.2) with time in the univariable analysis.

Two different sensitivity analyses were performed. First, same multivariable analysis was repeated after applying a square root transformation of mSASSS since its distribution is skewed. Second, because it is possible that missing radiographs during the follow up could cause a systemic bias, radiographic progression between two groups was repeatedly analyzed after multiple imputation of missing mSASSS using the Markov chain Monte Carlo method. In this analysis, mSASSS progression between the two groups was compared using an analysis of a covariance model with baseline mSASSS as a covariate.

All statistical analyses were done using IBM SPSS Statistics 20 (SPSS Inc., Chicago, IL, USA). P values <0.05 were considered statistically significant.

## Results

### Characteristics of the patients

Among 361 AS patients treated with etanercept or adalimumab in SNUH-biologics cohort, 110 patients with less than 2 years of TNFi treatment were excluded. Next, we excluded additional 86 patients because of lack of available radiographic sets (n = 81), total ankylosis of spine at baseline (n = 2) and >3 missing VCs (n = 3). Finally, a total of 165 patients were analyzed in the present study (S1 Fig).

Of the included patients, 86.1% were males, mean (S.D.) age was 39.6 (12.6) years and disease duration was 9.3 (6.7) years. Of 160 patients in whom HLA-B27 evaluation was performed, 147 (89.1%) were HLA-B27 positive. Mean (S.D.) duration of TNFi treatment was 4.9 (2.2) years. Etanercept was prescribed for 58 patients and the others used adalimumab throughout follow up. There were no significant differences in baseline characteristics such as age, gender, smoking status, HLA-B27 status, BASDAI, CRP and duration of TNFi treatment between included patients and excluded patients due to lack of available radiographs.

Demographic and clinical factors of the standard-dose group (n = 49) and the tapering group (n = 116) are described in Table 1. At the time of starting TNFi treatment, patients in the standard-dose group tended to be older (42.5 vs. 38.4 years, p = 0.057), were more likely to be current or ex-smokers (44.7% vs. 30.2%, p = 0.082), and showed higher BASDAI (7.1 vs. 6.3, p = 0.003) and more severe structural damage (17.3 vs. 11.9 mSASSS units, p = 0.059), all of which were possible predictors of syndesmophyte formation [25–27]. Other baseline statuses such as gender, BMI, serum CRP level and presence of hip involvement were not different between the two groups.

**Table 1:** Demographic and clinical features of the patients.

	All patients (n = 165)	Standard-dose group (n = 49)	Tapering group (n = 116)	*p* value ^a^
**Baseline (Starting TNFi treatment)**				
Age, mean (S.D.)	39.6 (12.6)	42.5 (13.2)	38.4 (12.2)	0.057
Male, n (%)	142 (86.1)	42 (85.7)	100 (86.2)	0.933
BMI, mean (S.D.)	23.2 (3.2)	23.1 (3.2)	23.2 (3.2)	0.837
Etanercept, n (%)	58 (35.2)	18 (36.7)	40 (34.5)	0.782
Disease duration in years, mean (S.D.)	9.3 (6.7)	9.3 (7.9)	9.2 (6.2)	0.983
HLA-B27 positive, n (%) ^b^	147 (91.9)	43 (87.8)	104 (93.7)	0.205
Current smoker, n (%) ^c^	46 (30.1)	17 (36.2)	29 (27.4)	0.273
Ever-smoker, n (%) ^c^	53 (34.6)	21 (44.7)	32 (30.2)	0.082
BASDAI, mean (S.D.)	6.5 (1.7)	7.1 (1.6)	6.3 (1.6)	0.003
CRP > 0.5mg/dL, n, (%)	119 (74.8)	35 (74.5)	84 (75.0)	0.944
Syndesmophyte at baseline, n (%)	68 (41.2)	27 (55.1)	41 (35.3)	0.018
Number of syndesmophyte at baseline, mean (S.D.)	2.8 (5.2)	3.8 (5.4)	2.4 (5.0)	0.107
Hip involvement at baseline, n (%)	45 (27.8)	13 (27.1)	32 (28.1)	0.898
Baseline mSASSS, mean (S.D.)	13.5 (16.6)	17.3 (17.7)	11.9 (16.0)	0.059
**During the TNFi treatment**				
Time-averaged CRP, mean (S.D.) ^d^	0.48 (0.44)	0.46 (0.41)	0.49 (0.45)	0.710
Time-averaged BASDAI, mean (S.D.) ^d^	2.1 (0.8)	2.4 (0.8)	1.9 (0.7)	<0.001
Concomitant NSAID, n (%)	123 (74.5)	40 (81.6)	83 (71.6)	0.174
High NSAID intake, n (%)	22 (13.3)	10 (20.4)	12 (10.3)	0.136

^a^ p Values for comparison between the standard-dose group and the tapering group

^b^ There were 5 missing values

^c^ There were 12 missing values

^d^ Mean value of those measured in the period between baseline and 45-month follow up

BASDAI, Bath Ankylosing Spondylitis Activity Index; CRP, C-reactive protein; HLA, human leukocyte antigen; NSAID, nonsteroidal antiinflammatory drug; S.D., standard deviation

Twenty-seven patients (55.1%) in the standard-dose group and forty-one (35.3%) patients in the tapering group had at least one syndesmophyte at baseline. This subgroup were older (46.4 vs. 34.8 years of age, p<0.001), had a longer disease duration (11.7 vs. 7.5 years, p<0.001) and a more hip involvement (38.5% vs. 20.6%, p = 0.013) than patients without baseline syndesmophytes. When a comparison between the two treatment strategies was performed in the syndesmophyte subgroup, the imbalance of the baseline and time-averaged BASDAI persisted but there were no significant differences in other clinical factors (S1 Table).

At 2- and 4-year of follow up, clinical features between the two groups were also comparable, except for older age and more severe structural damage in the standard-dose group at the 2-year follow up (S2 Table). During the observation period, 40 (81.6%) and 83 (71.6%) patients used concomitant NSAIDs in the standard-dose and the tapering group, respectively. Of them, 10 in the standard-dose group and 12 in the tapering group had a high NSAID intake. Time-averaged BASDAI over 4 years was also significantly higher in the standard-dose group, mainly due to the difference at baseline (2.4 vs. 1.9, p<0.001).

### Disease activity and dose tapering during follow up

**Fig 1:**
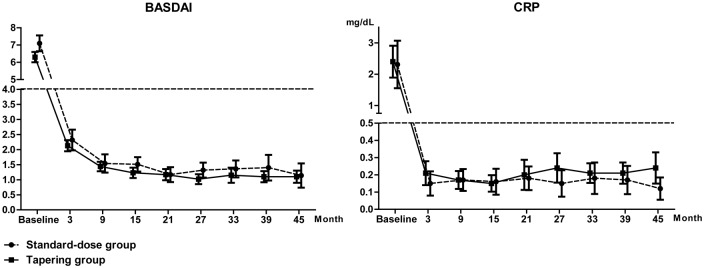
Disease activity as measured by BASDAI and serum CRP of the two treatment groups during the follow up. Error bars indicate 95% confidential intervals.

All included patients achieved low disease activity within 15 months of treatment and time to achieving low disease activity was similar between the two groups. Disease activity as measured by BASDAI and serum CRP was comparable between the two groups during the observation (Fig 1). In addition, this result was consistent when a comparison of disease activity between the two groups was performed in the syndesmophyte subgroup. All patients in the tapering group reduced the dose of TNFi after achieving low disease activity. The mean (S.D.) interval between starting TNFi and the first dose reduction was 36.6 (28.4) weeks and most patients (81.9%) in the tapering group reduced the dosage within a year. The mean (S.D.) proportion of the standard-dose period in total duration of TNFi treatment was 0.18 (0.15), which suggests that patients in the tapering group maintained a long-term reduced dose period after a relatively short standard-dose period for the induction of low disease activity.

The mean (S.D) TNFi index in the tapering group during the total observation period was 0.68 (0.13). It tended to continuously decrease during the first 2 years; the mean (S.D.) TNFi index in the period between baseline and six months was 0.95 (0.09), 0.72 (0.20) in 6~12 months, 0.64 (0.18) in 12~18 months and 0.60 (0.17) in 18~24 months (p<0.001). After 2 years, it did not change significantly over time (S2 Fig). Patients treated with etanercept showed a lower mean TNFi index than patients treated with adalimumab, although it was not statistically significant (0.59 vs. 0.65).

### Radiographic progression over time in the entire cohort

During follow up, 165, 137 and 81 radiographs were available at baseline, two years and four years, respectively. The number of patients who had complete sets of radiographs at baseline, 2- and 4-year follow up was 56 (33.9%) and their proportion was greater in the tapering group as compared with the standard-dose group (10/49, 20.4% vs. 46/116, 39.7%, p = 0.017). The mean (S.D.) interval between available radiographs was 2.4 (0.7) years.

Among various models of time, a linear time model showed the best fit for the data. At the entire group level, mSASSS progressed significantly over time, at a rate of 0.90 mSASSS units/year (p<0.001). Presence of syndesmophytes at baseline showed the most significant association with radiographic progression. Patients with baseline syndesmophytes had an approximately 4.5-fold higher rate of increase in mSASSS than patients without them (1.67 vs. 0.37 mSASSS units/year, p<0.001). In addition, radiographic progression occurred faster in patients older than 40 (1.29 vs. 0.59 mSASSS units/year), in longer (≥ 10 years) disease duration (1.08 vs. 0.80 mSASSS units/year), in ever-smokers (1.13 vs. 0.79 mSASSS units/year), in patients with hip involvement at baseline (1.20 vs. 0.78 mSASSS units/year) and in patients with higher (≥ 10 units) baseline mSASSS (1.57 vs. 0.44 mSASSS units/year). Gender, NSAID index, baseline and time-averaged disease activities and HLA-B27 had no significant interaction with time (S3 Table).

### Difference in radiographic progression between the two groups

**Fig 2:**
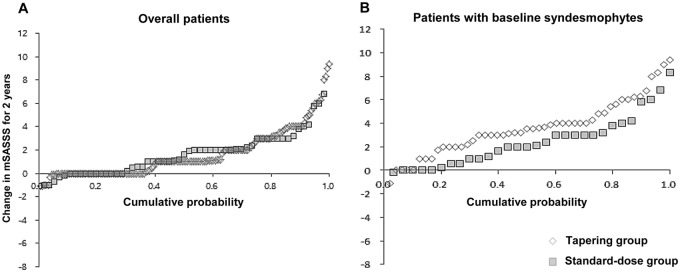
Cumulative probability plot of radiographic progression for two years in the entire cohort (A) in the subgroup of patients who had syndesmophytes at baseline (B). With baseline syndesmophytes, patients in the tapering group showed more rapid radiographic progression. All patients with an mSASSS available at each point independently of a two year progression were included; mSASSS, modified Stoke Ankylosing Spondylitis Spine Score.

At the entire group level, dosing strategy was not associated with radiographic progression over time. However, patients with baseline syndesmophytes in the tapering group showed significantly higher radiographic progression than patients with baseline syndesmophytes in the standard-dose group (interaction of time, baseline syndesmophyte and dosing strategy: p<0.001) (Fig 2). After stratification by the presence of baseline syndesmophytes, interaction between dosing strategy and time was significant only in patients with baseline syndesmophytes (β = 0.57, Bonferroni-corrected p = 0.004). Furthermore, in the subgroup analysis of patients with baseline syndesmophytes, the tapering group also showed a significantly more rapid progression compared to the standard-dose group (1.84 vs. 1.26 mSASSS units/year, p = 0.035). This result was consistent in the multivariable model which included smoking status (ever-smoker vs. non-smoker) as a covariate (1.72 vs. 1.23 mSASSS units/year, p = 0.023). Other clinical factors such as gender, disease duration, NSAID index, baseline and time-averaged disease activities and baseline mSASSS were not significantly associated with mSASSS progression over time in this subgroup (Table 2). On the other hand, a subgroup analysis of patients without baseline syndesmophytes showed similar radiographic progression irrespective of treatment regimen.

**Table 2:** Radiographic progression over time between the two dosing strategies in the presence of baseline syndesmophytes.

	Univariable analysis		Multivariable analysis ^b^	
	Regression coefficient (95% CI) ^a^ (n = 65–68)	*p* value	Regression coefficient (95% CI) (n = 65) ^c^	*p* value
Age	0.00 (-0.02 to 0.02)	0.941		
Male gender	0.42 (-0.44 to 1.28)	0.334		
BMI	0.04 (0.04 to 0.12)	0.281		
Disease duration	0.00 (-0.03 to 0.03)	0.973		
Ever-smoker	0.46 (-0.17 to 1.09)	0.148	0.49 (0.07 to 0.91)	0.025
Baseline BASDAI	0.08 (-0.06 to 0.23)	0.255		
HLA-B27	0.62 (-0.46 to 1.71)	0.258		
Baseline CRP (mg/dL)	0.06 (-0.04 to 0.16)	0.255		
Hip involvement	0.16 (-0.40 to 0.72)	0.564		
Baseline mSASSS	-0.01 (-0.02 to 0.01)	0.412		
Time-averaged CRP ^d^	0.22 (-0.47 to 0.91)	0.525		
Time-averaged BASDAI ^d^	-0.05 (-0.39 to 0.28)	0.749		
NSAID index	0.00 (-0.01 to 0.01)	0.888		
Dosing strategy	0.57 (0.04 to 1.10)	0.035	0.49 (0.07 to 0.91)	0.023
• Standard-dose group	1.26 (0.84 to 1.69)		1.23 (0.83 to 1.63)	
• Tapering group	1.84 (1.52 to 2.15)		1.72 (1.40 to 2.04)	

^a^ Regression coefficient indicates the progression of mSASSS over one year.

^b^ Any clinical factor which showed significant association (p <0.2) with mSASSS in the univariable analysis is included as a covariate.

^c^ There were three patients whose data on smoking status were missing.

^d^ Mean value of those measured in the period between baseline and 45-month follow up

BASDAI, Bath Ankylosing Spondylitis Activity Index; CI, confidential interval; CRP, C-reactive protein; HLA, human leukocyte antigen; NSAID, nonsteroidal antiinflammatory drug

Radiographic progression was further assessed by the number of newly developed syndesmophytes during follow up. Significant progression occurred only in patients who had baseline syndesmophytes, at a rate of 0.66/year (p<0.001). In this syndesmophyte subgroup, patients in the tapering group also showed a more rapidly increasing number of syndesmophytes after adjusting for the baseline number of syndesmophytes (0.73 vs. 0.52/year, p = 0.047). In contrast, patients without syndesmophytes at baseline showed little new bone formation over time, which was not significantly different between the two groups (0.07 vs. 0.06/year, p = 0.684).

### Reliability of assessing radiographic progression between the two readers

Inter-observer reliability of mSASSS was excellent, with an ICC (95% confidential interval) of 0.969 (0.958–0.976). The ICC for Inter-observer reliability for the change in mSASSS was 0.825 (0.715–0.895). In total of 383 sets of radiographs, major disagreement between two readers occurred in 28 (7.3%) cases and 23 (6.0%) were adjudicated by senior reader. SDC measured for all 2-year mSASSS progression was 1.3/2-year (S3 Fig). Among all observed 2-year mSASSS changes (n = 217), 93 (42.9%) progressions were above the SDC. In addition, there were 61 (28.1%) intervals which showed no change in mSASSS over time in both readers.

### Sensitivity analyses

**Fig 3:**
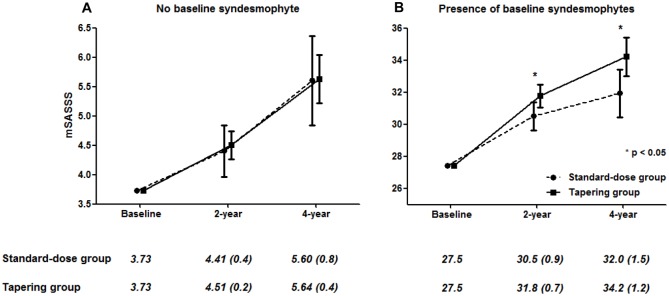
Radiographic progressions of the two treatment groups in patients without baseline syndesmophytes (A) and with baseline syndesmophytes (B). Missing data was replaced using multiple imputation. Values were given as means (standard error) and adjusted for baseline mSASSS. Error bars indicate 95% confidential intervals; mSASSS, modified Stoke Ankylosing Spondylitis Spine Score.

The results from the main analysis were not changed in the sensitivity analysis in which the mixed model was repeated after applying a square root transformation of mSASSS. In addition, the second sensitivity analysis based on multiple imputation of missing radiographic data led to concordant results for the effect of dose tapering on radiographic progression (Fig 3). Finally, to investigate the impact of measurement errors on the main result, we performed identical mixed model analysis after excluding 63 progressions which showed changes in mSASSS over time but with a rate lower than SDC. In this analysis, the interaction of time, baseline syndesmophyte and dosing strategy was significant (β = 1.47, standard error = 0.65, p = 0.026), along with the main analysis. After stratification by the presence of baseline syndesmophytes, the tapering group showed significantly more rapid radiographic progression than the standard-dose group but only in the presence of baseline syndesmophytes (β = 1.02, Bonferroni-corrected p = 0.038).

## Discussion

In the present study, we investigated the radiographic progression over four years of TNFi treatment and associated clinical factors in patients with AS. Along with previous studies, radiographic progression followed a linear course at the group level (0.9 mSASSS units/year). Rapid radiographic progression was significantly associated with longer disease duration, older age, ever-smoking, hip involvement and most importantly, baseline structural damage.

The rate of radiographic progression in this study was higher than previous phase III studies of TNFi where change in mSASSS over time was approximately 1 unit/2 years. This conflicting result is explained by the difference in the methodology regarding mSASSS scoring. In the present study, readers were not blinded to the time sequence of radiographs (chronological scoring) because it is more sensitive to change than reading without information on the time sequence (paired scoring). For example, in a previous study which investigated difference in mSASSS progression between two scoring methods in OASIS cohort, chronological reading showed significantly more rapid progression than paired reading at 2 years (2.1 vs. 1.0 unit) and this progression rate was comparable to that of our study [23].

To our knowledge, our study is the first to describe the impact of dose tapering of TNFi on radiographic progression in AS. Without baseline syndesmophytes, the rate of mSASSS progression was generally negligible and was not different between the two strategies. However, in patients who had syndesmophytes before initiating TNFi, dose tapering was significantly associated with rapid radiographic progression measured by both mSASSS and the number of newly developed syndesmophytes. This result is of particular interest because it provides insight in selecting patients who can benefit from the dose tapering strategy without structural progression. In general, AS patients who started TNFi should continue the treatment, since its discontinuation will usually lead to clinical relapse [28]. However, long-term use of TNFi is associated with increased risk of adverse events and a substantial burden of cost to patients [11, 13]. Therefore, dose reduction of TNFi seems to be ideal regarding its favorable efficacy and significantly low cost [14]. But for the implementation of this strategy in AS, precise analysis regarding cost-effectiveness is mandatory in advance, and radiographic progression is an important component to estimate efficacy of the treatment. In previous studies, a favorable clinical response to TNFi was the sole indication for dose reduction [14, 15, 19, 29]. However, AS patients who started TNFi mostly showed rapid symptomatic improvement and new bone formation in AS is known to be persistent irrespective of the clinical disease status [30]. Therefore, clinical disease activity alone may not be an ideal tool to decide dose tapering of TNFi in AS. By contrast, presence of syndesmophytes when initiating TNFi can effectively predict further structural damage induced by dose tapering and be easily identified in routine clinical practice. Unfortunately, this study did not investigate subsequent functional impairment and loss of quality of life which can result from radiographic progression because a four-year timeframe may be too short to detect a significant change. Functional impairment and loss of quality of life may be evaluated further in future studies with longer follow up periods.

The mechanism underlying rapid radiographic progression accompanying dose tapering in patients with baseline syndesmophyte remains uncertain. Previous studies using MRI suggested that VC inflammation was clearly associated with future radiographic progression in patients with AS [5, 31]. Although TNFi improves inflammatory lesions effectively, some of them persisted after the two years of treatment [32, 33]. Therefore, it is possible that remaining lesions triggered by inadequate anti-inflammation could progress to the advanced structural changes such as fat disposition [34, 35]. Interestingly, no difference in radiographic progression according to dosing strategies appeared in patients without baseline syndesmophytes. Since they had a relatively shorter disease duration, it suggests that early TNFi treatment has a potential role in preventing structural progression in AS [10, 34]. However, the exact relationship between dose tapering of TNFi and the dynamics of MRI VC lesions needs to be investigated in future studies.

It is also interesting that concomitant NSAID use and high NSAID intake did not have a significant interaction with mSASSS progression. Although previous clinical studies indicated the disease-modifying effect of NSAIDs, a recent randomized controlled trial (ENRADAS) failed to show this in patients with continuous NSAIDs [21, 36, 37]. As in ENRADAS, there were relatively few patients who had used selected inhibitors of cyclooxygenase-2 (coxibs) in this study (9/123), so it is likely that superior inhibitory effect on new bone formation of coxibs may have caused this conflicting result [38].

This study has several limitations to be considered. First, this study is mainly a retrospective design and there was no fixed protocol for the tapering of TNFi. Furthermore, although dose reduction was attempted after achieving low disease activity and duration of prior standard-dose treatment was relatively homogeneous, we cannot exclude the possibility that some unmeasured confounders such as physicians’ preference for tapering strategy influenced the selection of patients at reduced dosage. However, disease activity was comparable between the two groups during the observation. In addition, the government health care system in South Korea reimburses all AS patients for 90% of related medication costs. Therefore, it is less likely that patients’ economic status was a major determinant in deciding treatment strategy in this study. Second, there were considerable missing radiographs, especially at 4-year follow up. Although a mixed model analysis statistically handles missing data effectively, it could cause a systemic bias. Furthermore, we did not perform analyses restricted to patients with a complete set of sequential radiographs because their number in the standard-dose group was too small for statistical analysis. Although a sensitivity analysis in which comparison of radiographic progression was performed after multiple imputation did not alter the result, it should be reproduced in future studies. Finally, baseline characteristics between the two groups were rather different since the treatment strategies were not randomly assigned to patients. However, patients in the standard-dose group had slightly greater proportion of parameters predicting radiographic progression such as disease activity, smoking status and baseline mSASSS. Therefore, it is less likely that these disparities severely biased the results in this study.

In conclusion, the results of the present study suggest that a dose tapering strategy of TNFi in the treatment of AS is associated with rapid radiographic progression in the presence of baseline syndesmophytes. Although this finding needs to be confirmed in future studies with longer follow up periods, it may have a substantial impact on establishing universal recommendations for a dose tapering strategy of TNFi in AS.

## Supporting Information

S1 Fig Flowchart of the patients. (TIF)

S2 Fig Longitudinal change of TNFi index in the tapering group during the entire follow up. (TIF)

S3 Fig Bland-Altman plot presenting intra-observer reliability of 2-year mSASSS change. (TIF)

S1 Table Demographic and clinical features of the patients stratified by the presence of baseline syndesmophytes. (DOCX)

S2 Table Demographic and clinical features of patients at 2- and 4-year of follow up. (DOCX)

S3 Table Effect of clinical features on radiographic progression over time. (DOCX)

## References

[1] Machado P, Landewe R, Braun J, Hermann KG, Baker D, van der Heijde D. Both structural damage and inflammation of the spine contribute to impairment of spinal mobility in patients with ankylosing spondylitis. Annals of the rheumatic diseases. 2010;69(8):1465–70. 10.1136/ard.2009.124206 20498215

[2] Machado P, Landewe R, Braun J, Hermann KG, Baraliakos X, Baker D, et al A stratified model for health outcomes in ankylosing spondylitis. Annals of the rheumatic diseases. 2011;70(10):1758–64. 10.1136/ard.2011.150037 21791453

[3] Landewe R, Dougados M, Mielants H, van der Tempel H, van der Heijde D. Physical function in ankylosing spondylitis is independently determined by both disease activity and radiographic damage of the spine. Annals of the rheumatic diseases. 2009;68(6):863–7. 10.1136/ard.2008.091793 18628283

[4] Braun J, van den Berg R, Baraliakos X, Boehm H, Burgos-Vargas R, Collantes-Estevez E, et al 2010 update of the ASAS/EULAR recommendations for the management of ankylosing spondylitis. Annals of the rheumatic diseases. 2011;70(6):896–904. 10.1136/ard.2011.151027 21540199 PMC3086052

[5] Braun J, Landewe R, Hermann KG, Han J, Yan S, Williamson P, et al Major reduction in spinal inflammation in patients with ankylosing spondylitis after treatment with infliximab: results of a multicenter, randomized, double-blind, placebo-controlled magnetic resonance imaging study. Arthritis and rheumatism. 2006;54(5):1646–52. 10.1002/art.21790 16646033

[6] van der Heijde D, Landewe R, Baraliakos X, Houben H, van Tubergen A, Williamson P, et al Radiographic findings following two years of infliximab therapy in patients with ankylosing spondylitis. Arthritis and rheumatism. 2008;58(10):3063–70. 10.1002/art.23901 18821688

[7] van der Heijde D, Landewe R, Einstein S, Ory P, Vosse D, Ni L, et al Radiographic progression of ankylosing spondylitis after up to two years of treatment with etanercept. Arthritis and rheumatism. 2008;58(5):1324–31. Epub 2008/04/29. 10.1002/art.23471 18438853

[8] van der Heijde D, Salonen D, Weissman BN, Landewe R, Maksymowych WP, Kupper H, et al Assessment of radiographic progression in the spines of patients with ankylosing spondylitis treated with adalimumab for up to 2 years. Arthritis research & therapy. 2009;11(4):R127. 19703304 10.1186/ar2794 PMC2745811

[9] Baraliakos X, Haibel H, Listing J, Sieper J, Braun J. Continuous long-term anti-TNF therapy does not lead to an increase in the rate of new bone formation over 8 years in patients with ankylosing spondylitis. Annals of the rheumatic diseases. 2014;73(4):710–5. 10.1136/annrheumdis-2012-202698 23505240

[10] Haroon N, Inman RD, Learch TJ, Weisman MH, Lee M, Rahbar MH, et al The impact of tumor necrosis factor alpha inhibitors on radiographic progression in ankylosing spondylitis. Arthritis and rheumatism. 2013;65(10):2645–54. 10.1002/art.38070 23818109 PMC3974160

[11] Schabert VF, Watson C, Joseph GJ, Iversen P, Burudpakdee C, Harrison DJ. Costs of tumor necrosis factor blockers per treated patient using real-world drug data in a managed care population. Journal of managed care pharmacy: JMCP. 2013;19(8):621–30. Epub 2013/10/01. 10.18553/jmcp.2013.19.8.621 24074008 PMC10438048

[12] Ara RM, Reynolds AV, Conway P. The cost-effectiveness of etanercept in patients with severe ankylosing spondylitis in the UK. Rheumatology (Oxford). 2007;46(8):1338–44. Epub 2007/06/08. 17553909 10.1093/rheumatology/kem133

[13] Baddley JW, Winthrop KL, Chen L, Liu L, Grijalva CG, Delzell E, et al Non-viral opportunistic infections in new users of tumour necrosis factor inhibitor therapy: results of the SAfety Assessment of Biologic ThERapy (SABER) study. Annals of the rheumatic diseases. 2014;73(11):1942–8. 10.1136/annrheumdis-2013-203407 23852763 PMC4273901

[14] Zavada J, Uher M, Sisol K, Forejtova S, Jarosova K, Mann H, et al A tailored approach to reduce dose of anti-TNF drugs may be equally effective, but substantially less costly than standard dosing in patients with ankylosing spondylitis over 1 year: a propensity score-matched cohort study. Annals of the rheumatic diseases. 2016;75(1):96–102. 10.1136/annrheumdis-2014-205202 25165033

[15] Paccou J, Bacle-Boutry MA, Solau-Gervais E, Bele-Philippe P, Flipo RM. Dosage adjustment of anti-tumor necrosis factor-alpha inhibitor in ankylosing spondylitis is effective in maintaining remission in clinical practice. The Journal of rheumatology. 2012;39(7):1418–23. Epub 2012/06/19. 10.3899/jrheum.111337 22707611

[16] Bruynesteyn K, Boers M, Kostense P, van der Linden S, van der Heijde D. Deciding on progression of joint damage in paired films of individual patients: smallest detectable difference or change. Annals of the rheumatic diseases. 2005;64(2):179–82. 10.1136/ard.2003.018457 15286006 PMC1755378

[17] van der Linden S, Valkenburg HA, Cats A. Evaluation of diagnostic criteria for ankylosing spondylitis. A proposal for modification of the New York criteria. Arthritis and rheumatism. 1984;27(4):361–8. Epub 1984/04/01. 6231933 10.1002/art.1780270401

[18] van der Heijde D, Sieper J, Maksymowych WP, Dougados M, Burgos-Vargas R, Landewe R, et al 2010 Update of the international ASAS recommendations for the use of anti-TNF agents in patients with axial spondyloarthritis. Annals of the rheumatic diseases. 2011;70(6):905–8. 10.1136/ard.2011.151563 21540200

[19] Navarro-Compan V, Moreira V, Ariza-Ariza R, Hernandez-Cruz B, Vargas-Lebron C, Navarro-Sarabia F. Low doses of etanercept can be effective in ankylosing spondylitis patients who achieve remission of the disease. Clinical rheumatology. 2011;30(7):993–6. Epub 2011/03/05. 10.1007/s10067-011-1722-5 21373780

[20] Dougados M, Simon P, Braun J, Burgos-Vargas R, Maksymowych WP, Sieper J, et al ASAS recommendations for collecting, analysing and reporting NSAID intake in clinical trials/epidemiological studies in axial spondyloarthritis. Annals of the rheumatic diseases. 2011;70(2):249–51. 10.1136/ard.2010.133488 20829199

[21] Poddubnyy D, Rudwaleit M, Haibel H, Listing J, Marker-Hermann E, Zeidler H, et al Effect of non-steroidal anti-inflammatory drugs on radiographic spinal progression in patients with axial spondyloarthritis: results from the German Spondyloarthritis Inception Cohort. Annals of the rheumatic diseases. 2012;71(10):1616–22. 10.1136/annrheumdis-2011-201252 22459541

[22] Creemers MC, Franssen MJ, van't Hof MA, Gribnau FW, van de Putte LB, van Riel PL. Assessment of outcome in ankylosing spondylitis: an extended radiographic scoring system. Annals of the rheumatic diseases. 2005;64(1):127–9. 10.1136/ard.2004.020503 15051621 PMC1755183

[23] Wanders A, Landewe R, Spoorenberg A, de Vlam K, Mielants H, Dougados M, et al Scoring of radiographic progression in randomised clinical trials in ankylosing spondylitis: a preference for paired reading order. Annals of the rheumatic diseases. 2004;63(12):1601–4. 10.1136/ard.2004.022038 15297280 PMC1754835

[24] West BT. Analyzing longitudinal data with the linear mixed models procedure in SPSS. Evaluation & the health professions. 2009;32(3):207–28. 19679634 10.1177/0163278709338554

[25] Poddubnyy D, Haibel H, Listing J, Marker-Hermann E, Zeidler H, Braun J, et al Baseline radiographic damage, elevated acute-phase reactant levels, and cigarette smoking status predict spinal radiographic progression in early axial spondylarthritis. Arthritis and rheumatism. 2012;64(5):1388–98. 10.1002/art.33465 22127957

[26] Ramiro S, van der Heijde D, van Tubergen A, Stolwijk C, Dougados M, van den Bosch F, et al Higher disease activity leads to more structural damage in the spine in ankylosing spondylitis: 12-year longitudinal data from the OASIS cohort. Annals of the rheumatic diseases. 2014;73(8):1455–61. 10.1136/annrheumdis-2014-205178 24812292

[27] Maas F, Spoorenberg A, Brouwer E, Bos R, Efde M, Chaudhry RN, et al Spinal radiographic progression in patients with ankylosing spondylitis treated with TNF-alpha blocking therapy: a prospective longitudinal observational cohort study. PloS one. 2015;10(4):e0122693 10.1371/journal.pone.0122693 25879956 PMC4400173

[28] Brandt J, Khariouzov A, Listing J, Haibel H, Sorensen H, Grassnickel L, et al Six-month results of a double-blind, placebo-controlled trial of etanercept treatment in patients with active ankylosing spondylitis. Arthritis and rheumatism. 2003;48(6):1667–75. Epub 2003/06/10. 10.1002/art.11017 12794835

[29] Lee SH, Lee YA, Hong SJ, Yang HI. Etanercept 25 mg/week is effective enough to maintain remission for ankylosing spondylitis among Korean patients. Clinical rheumatology. 2008;27(2):179–81. Epub 2007/09/18. 10.1007/s10067-007-0674-2 17874173

[30] Ramiro S, Stolwijk C, van Tubergen A, van der Heijde D, Dougados M, van den Bosch F, et al Evolution of radiographic damage in ankylosing spondylitis: a 12 year prospective follow-up of the OASIS study. Annals of the rheumatic diseases. 2015;74(1):52–9. 10.1136/annrheumdis-2013-204055 23956249

[31] Lambert RG, Salonen D, Rahman P, Inman RD, Wong RL, Einstein SG, et al Adalimumab significantly reduces both spinal and sacroiliac joint inflammation in patients with ankylosing spondylitis: a multicenter, randomized, double-blind, placebo-controlled study. Arthritis and rheumatism. 2007;56(12):4005–14. 10.1002/art.23044 18050198

[32] Sieper J, Baraliakos X, Listing J, Brandt J, Haibel H, Rudwaleit M, et al Persistent reduction of spinal inflammation as assessed by magnetic resonance imaging in patients with ankylosing spondylitis after 2 yrs of treatment with the anti-tumour necrosis factor agent infliximab. Rheumatology (Oxford). 2005;44(12):1525–30 16091396 10.1093/rheumatology/kei046

[33] Baraliakos X, Davis J, Tsuji W, Braun J. Magnetic resonance imaging examinations of the spine in patients with ankylosing spondylitis before and after therapy with the tumor necrosis factor alpha receptor fusion protein etanercept. Arthritis and rheumatism. 2005;52(4):1216–23. 10.1002/art.20977 15818694

[34] Maksymowych WP, Morency N, Conner-Spady B, Lambert RG. Suppression of inflammation and effects on new bone formation in ankylosing spondylitis: evidence for a window of opportunity in disease modification. Annals of the rheumatic diseases. 2013;72(1):23–8. 10.1136/annrheumdis-2011-200859 22562977

[35] Machado PM, Baraliakos X, van der Heijde D, Braun J, Landewe R. MRI vertebral corner inflammation followed by fat deposition is the strongest contributor to the development of new bone at the same vertebral corner: a multilevel longitudinal analysis in patients with ankylosing spondylitis. Annals of the rheumatic diseases. 2015. 10.1136/annrheumdis-2015-208011 26462728

[36] Wanders A, Heijde D, Landewe R, Behier JM, Calin A, Olivieri I, et al Nonsteroidal antiinflammatory drugs reduce radiographic progression in patients with ankylosing spondylitis: a randomized clinical trial. Arthritis and rheumatism. 2005;52(6):1756–65. 10.1002/art.21054 15934081

[37] Sieper J, Listing J, Poddubnyy D, Song IH, Hermann KG, Callhoff J, et al Effect of continuous versus on-demand treatment of ankylosing spondylitis with diclofenac over 2 years on radiographic progression of the spine: results from a randomised multicentre trial (ENRADAS). Annals of the rheumatic diseases. 2015. d 10.1136/annrheumdis-2015-207897 26242443

[38] Vuolteenaho K, Moilanen T, Moilanen E. Non-steroidal anti-inflammatory drugs, cyclooxygenase-2 and the bone healing process. Basic & clinical pharmacology & toxicology. 2008;102(1):10–4. 17973900 10.1111/j.1742-7843.2007.00149.x

