# Supplementary figures and images for: Impact of Dose Tapering of Tumor Necrosis Factor Inhibitor on Radiographic Progression in Ankylosing Spondylitis
Source: PLoS One. 2016 Dec 29;11(12):e0168958. doi: 10.1371/journal.pone.0168958 (PMC5199008; doi:10.1371/journal.pone.0168958)

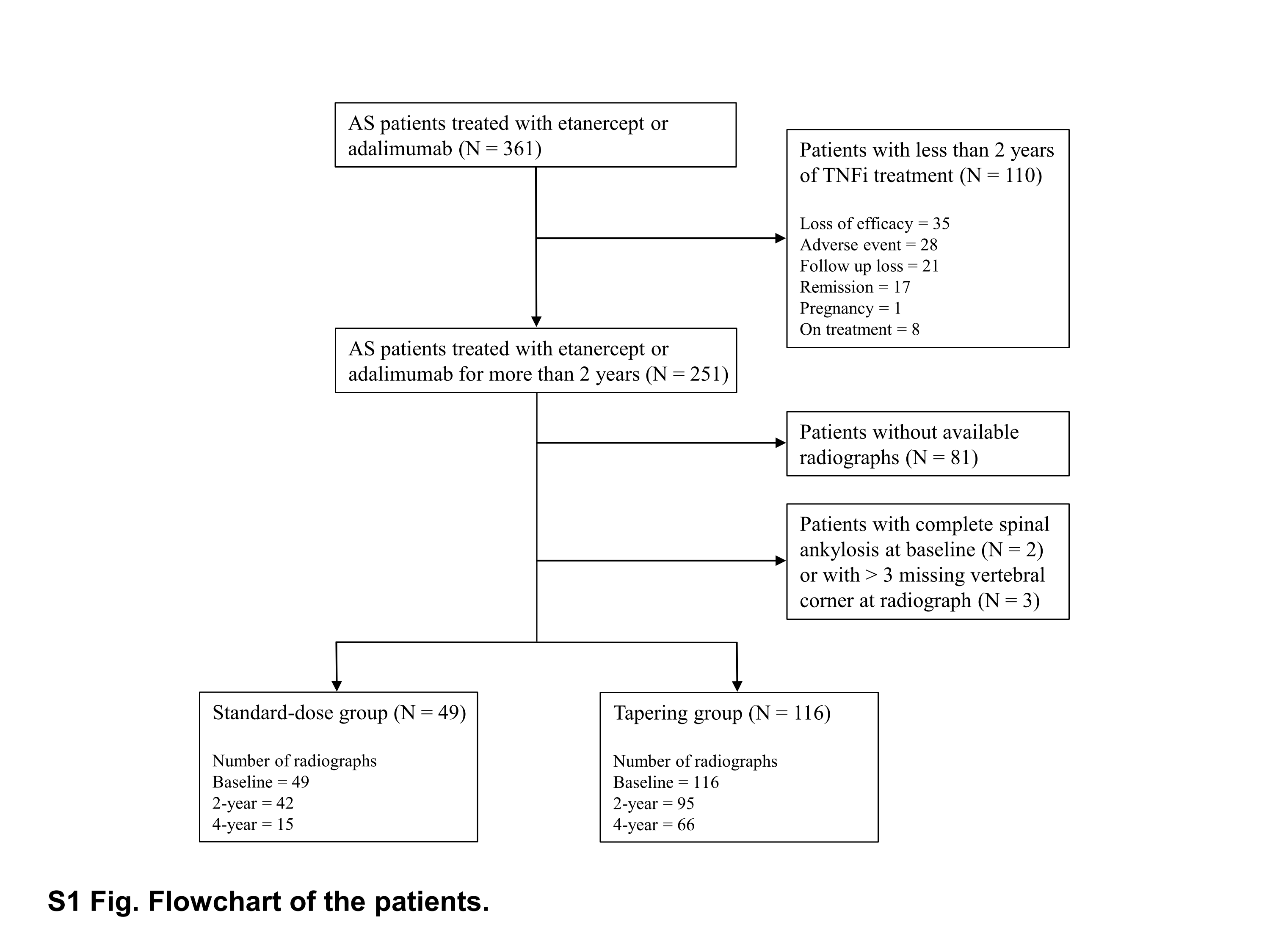

Supplement: S1 Fig — (TIF) [file pone.0168958.s001.tif]

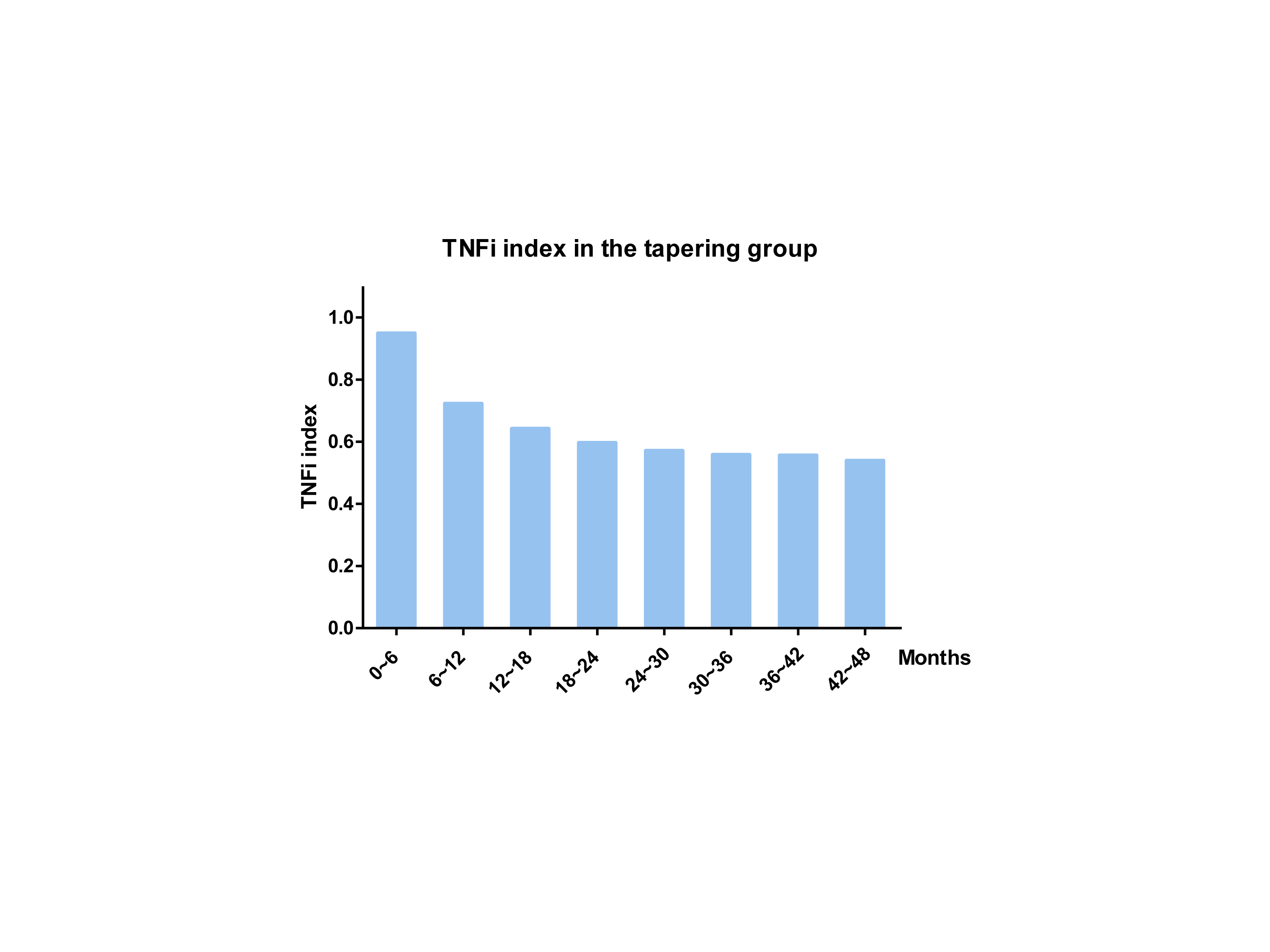

Supplement: S2 Fig — (TIF) [file pone.0168958.s002.tif]

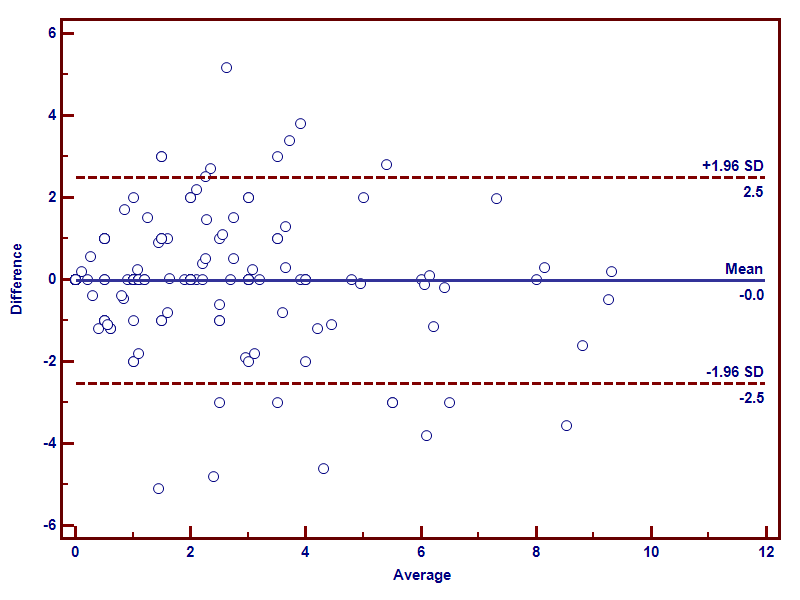

Supplement: S3 Fig — (TIF) [file pone.0168958.s003.tif]
