# Supplementary material for: Impact of Dose Tapering of Tumor Necrosis Factor Inhibitor on Radiographic Progression in Ankylosing Spondylitis
Source: PLoS One. 2016 Dec 29;11(12):e0168958. doi: 10.1371/journal.pone.0168958 (PMC5199008; doi:10.1371/journal.pone.0168958)
Supplement: S1 Table — (DOCX) [file pone.0168958.s004.docx]

**S1 Table.** **Demographic and clinical features of the patients stratified by the presence of baseline syndesmophytes**

|  | No baseline syndesmophyte | | |  | Baseline syndesmophytes | | |
| --- | --- | --- | --- | --- | --- | --- | --- |
|  | Standard-dose group (N=22) | Tapering group (N=75) | p Value |  | Standard-dose group (N=27) | Tapering group (N=41) | p Value |
| **Baseline (Starting TNFi treatment)** |  |  |  |  |  |  |  |
| Age, mean (S.D.) ^a^ | 38.3 (14.9) | 33.8 (9.6) | 0.187 |  | 45.8 (10.8) | 46.8 (11.9) | 0.729 |
| Male, n (%) | 20 (90.9%) | 64 (85.3%) | 0.726 |  | 22 (81.5%) | 36 (87.8%) | 0.502 |
| BMI, mean (S.D.) | 22.3 (3.4) | 23.0 (3.1) | 0.353 |  | 23.7 (3.0) | 23.6 (3.5) | 0.856 |
| Etanercept, n (%) | 8 (36.4%) | 26 (34.7%) | 0.883 |  | 10 (37.0%) | 14 (34.1%) | 0.807 |
| Disease duration in years, mean (S.D.) ^a^ | 6.5 (5.0) | 7.8 (4.2) | 0.239 |  | 11.5 (9.0) | 11.9 (8.1) | 0.866 |
| HLA-B27 positive, n (%) | 18 (81.8%) | 66 (93.0%) | 0.208 |  | 25 (92.6%) | 38 (95.0%) | 0.683 |
| Current smoker, n (%) | 6 (30.0%) | 15 (21.7 %) | 0.464 |  | 11 (40.7%) | 14 (36.8%) | 0.750 |
| Current or ex-smoker, n (%) | 7 (35.0%) | 18 (26.5%) | 0.457 |  | 14 (51.9%) | 14 (36.8%) | 0.228 |
| BASDAI, mean (S.D.) | 7.2 (1.8) | 6.4 (1.4) | 0.055 |  | 7.1 (1.5) | 6.0 (2.0) | 0.028 |
| CRP > 0.5mg/dL, n, (%) | 15 (75.0%) | 51 (70.8%) | 0.787 |  | 20 (74.1%) | 33 (82.5%) | 0.405 |
| Hip involvement at baseline, n (%) ^a^ | 2 (9.1%) | 18 (24.0%) | 0.129 |  | 11 (42.3%) | 14 (35.9%) | 0.603 |
| Baseline mSASSS, mean (S.D.) ^a^ | 4.7 (6.3) | 3.4 (4.3) | 0.275 |  | 27.5 (17.4) | 27.4 (18.0) | 0.994 |
| **During the TNFi treatment** |  |  |  |  |  |  |  |
| Time-averaged CRP, mean (S.D.) ^b^ | 0.57 (0.52) | 0.43 (0.45) | 0.209 |  | 0.36 (0.26) | 0.46 (0.26) | 0.124 |
| Time-averaged BASDAI, mean (S.D.) ^b^ | 2.3 (0.8) | 1.9 (0.6) | 0.013 |  | 2.4 (0.9) | 1.9 (0.8) | 0.023 |
| Concomitant NSAID, n (%) | 16 (72.7%) | 52 (69.3%) | 0.760 |  | 24 (88.9%) | 31 (75.6%) | 0.219 |
| High NSAID intake, n (%) | 5 (22.7%) | 6 (8.0%) | 0.158 |  | 5 (18.5%) | 6 (14.6%) | 0.392 |

^a^ p < 0.05 between subgroup of patients with and without baseline syndesmophytes

^b^ Mean value of those measured in the period between the baseline and 45-month follow up

BAS.D.AI, Bath Ankylosing Spondylitis Activity Index; CRP, C-reactive protein; NSAID, nonsteroidal antiinflammatory drug; S.D., Standard deviation
