# Supplementary material for: Impact of Dose Tapering of Tumor Necrosis Factor Inhibitor on Radiographic Progression in Ankylosing Spondylitis
Source: PLoS One. 2016 Dec 29;11(12):e0168958. doi: 10.1371/journal.pone.0168958 (PMC5199008; doi:10.1371/journal.pone.0168958)
Supplement: S2 Table — (DOCX) [file pone.0168958.s005.docx]

**S2 Table.** **Demographic and clinical features of patients at 2- and 4-year follow up period.**

|  | 2-year follow up (n=137) | | |  | 4-year follow up (n=81) | | |
| --- | --- | --- | --- | --- | --- | --- | --- |
|  | Standard-dose group (N=42) | Tapering group (N=95) | *p* value |  | Standard-dose group (N=16) | Tapering group (N=65) | *p* value |
| Age, mean (S.D.) | 44.1 (13.7) | 38.3 (11.1) | 0.009 |  | 45.6 (11.7) | 44.5 (12.7) | 0.739 |
| Male, n (%) | 36 (85.7%) | 84 (88.4%) | 0.658 |  | 16 (100.0%) | 57 (87.7%) | 0.139 |
| Disease duration in years, mean (S.D.) | 10.9 (7.8) | 10.4 (4.9) | 0.705 |  | 15.6 (8.0) | 14.7 (6.5) | 0.658 |
| HLA-B27 positive, n (%) | 36 (85.7%) | 84 (92.3%) | 0.234 |  | 14 (87.5%) | 59 (95.2%) | 0.265 |
| BASDAI, mean (S.D.) | 1.2 (0.9) | 1.0 (0.8) | 0.249 |  | 0.8 (0.6) | 1.1 (0.8) | 0.268 |
| CRP (mg/dL), mean (S.D.) | 0.1 (0.2) | 0.2 (0.4) | 0.132 |  | 0.1 (0.2) | 0.3 (0.4) | 0.304 |
| CRP > 0.5mg/dL, n (%) | 2 (5.3%) | 13 (14.1%) | 0.150 |  | 3 (20.0%) | 9 (14.5%) | 0.599 |
| mSASSS, mean (S.D.) | 18.9 (18.6) | 11.8 (16.1) | 0.024 |  | 21.0 (18.0) | 16.1 (19.1) | 0.367 |
| Presence of syndesmophytes, n (%) | 24 (57.1%) | 33 (34.7%) | 0.014 |  | 11 (68.8%) | 28 (43.1%) | 0.094 |

BASDAI, Bath Ankylosing Spondylitis Activity Index; CRP, C-reactive protein; S.D., Standard deviation
