# Supplementary material for: Impact of Dose Tapering of Tumor Necrosis Factor Inhibitor on Radiographic Progression in Ankylosing Spondylitis
Source: PLoS One. 2016 Dec 29;11(12):e0168958. doi: 10.1371/journal.pone.0168958 (PMC5199008; doi:10.1371/journal.pone.0168958)
Supplement: S3 Table — (DOCX) [file pone.0168958.s006.docx]

**S3 Table. Effect of clinical features on radiographic progression over time**

|  | Univariable regression coefficient (SE) ^a^ | *p* value ^b^ | N |
| --- | --- | --- | --- |
| Time (year) | 0.90 (0.07) | <0.001 | 165 |
| Time x Disease duration |  | 0.001 | 165 |
| Progression in disease duration < 10 years | 0.80 (0.09) |  | 110 |
| Progression in disease duration ≥ 10 years | 1.08 (0.12) |  | 55 |
| Time x Age |  | <0.001 | 165 |
| Progression in patient’s age < 40 | 0.59 (0.09) |  | 95 |
| Progression in patient’s age ≥ 40 | 1.29 (0.10) |  | 70 |
| Time x Smoking |  | 0.026 | 153 |
| Progression in non-smoker | 0.80 (0.09) |  | 100 |
| Progression in ever-smoker | 1.18 (0.14) |  | 53 |
| Time x Hip involvement |  | 0.010 | 162 |
| Progression in patients with a hip involvement | 0.78 (0.08) |  | 117 |
| Progression in patients without a hip involvement | 1.20 (0.14) |  | 45 |
| Time x baseline mSASSS |  | <0.001 | 165 |
| Progression when baseline mSASSS < 10 unit | 0.44 (0.08) |  | 99 |
| Progression when baseline mSASSS ≥ 10 unit | 1.57 (0.10) |  | 66 |
| Time x baseline syndesmophytes |  | <0.001 | 165 |
| Progression in patients without baseline syndesmophytes | 0.37 (0.07) |  | 97 |
| Progression in patients with baseline syndesmophytes | 1.67 (0.10) |  | 68 |
| Time x Gender |  | 0.887 |  |
| Time x BMI |  | 0.205 |  |
| Time x HLA-B27 |  | 0.217 |  |
| Time x baseline BASDAI |  | 0.369 |  |
| Time x baseline CRP |  | 0.793 |  |
| Time x time-averaged BASDAI |  | 0.665 |  |
| Time x time-averaged CRP |  | 0.179 |  |
| Time x NSAID index |  | 0.110 |  |
| Time x Dosing strategy |  | 0.890 |  |
| Time x Baseline syndesmophytes x Dosing strategy | 1.32 (0.14) | < 0.001 | 165 |
| Time x Dosing strategy in patients without baseline syndesmophytes | -0.02 (0.18) | 1.000 ^c^ | 97 |
| Time x Dosing strategy in patients with baseline syndesmophytes | 0.57 (0.19) | 0.004 ^c^ | 68 |

^a^ Indicates the progression of mSASSS over one year

^b^ p Values are shown for the interaction between time and each clinical factor. In the presence of the significant interaction, regression coefficient is calculated after stratification into subgroups

^C^ Bonferroni adjustements for *p* values were performed

BASDAI, Bath Ankylosing Spondylitis Activity Index; CRP, C-reactive protein; HLA, human leukocyte antigen; NSAID, nonsteroidal aniinflammatory drug; SE, Standard error
